# Sex-dependent modulation of acute respiratory distress syndrome by *Bacteroides acidifaciens*: gut microbiome impact on lung inflammation

**DOI:** 10.3389/fimmu.2025.1653309

**Published:** 2025-09-22

**Authors:** Shanieka Staley, Virginia Walkup, Stacey Oxendine, Zannatul Mauya, Jordan Williams, Philip Brandon Busbee, Kiesha Wilson

**Affiliations:** Department of Pathology, Microbiology, and Immunology, University of South Carolina School of Medicine, Columbia, SC, United States

**Keywords:** Bacteroides acidifaciens, acute respiratory distress syndrome (ARDS), sex differences, gut-lung axis, microbiome-host interactions

## Abstract

*Bacteroides acidifaciens* (BA), a common gut commensal, is known to modulate immune responses, but its role in acute respiratory distress syndrome (ARDS) and potential sex-specific effects remain poorly understood. To investigate this, male and female mice were colonized with BA prior to induction of ARDS using dual doses of staphylococcal enterotoxin B (SEB), a potent superantigen that triggers cytokine storm–driven lung injury. Clinical parameters, histopathology, gene expression, ELISA, flow cytometry, and gut barrier assessments were used to evaluate outcomes. BA pre-treatment significantly improved lung function, and attenuated pulmonary inflammation in male mice, correlating with increased IL-22, expansion of γδ T cells, and upregulation of colonic tight junction proteins. In contrast, BA exacerbated ARDS symptoms in females, increasing Th17 responses, neutrophil infiltration, and IgA-associated immune activation while impairing gut barrier integrity. These findings reveal that BA exerts divergent, sex-dependent effects in ARDS, highlighting the critical need to consider sex as a biological variable in microbiome-based therapies targeting inflammatory lung disease.

## 1 Introduction

Acute respiratory distress syndrome (ARDS) is a life-threatening condition characterized by severe lung inflammation, increased alveolar permeability, and impaired gas exchange (1, 2). It is commonly triggered by infections, trauma, or sepsis and carries a high risk of morbidity and mortality (3). The COVID-19 pandemic has underscored the devastating impact of ARDS, as severe cases of SARS-CoV-2 infection frequently progress to ARDS, significantly contributing to hospitalizations and fatalities (4). Studies of COVID-19-associated ARDS have revealed widespread alterations in the gut and lung microbiomes, with dysbiosis linked to immune dysfunction and worsened clinical outcomes (5, 6). These observations highlight the urgent need to investigate microbial influences on inflammation and disease severity in ARDS.

Although advances in supportive care have improved survival, effective targeted therapies for ARDS remain elusive (7). Recently, the gut-lung axis has emerged as a critical pathway in regulating pulmonary immunity. Disruptions in gut microbiota composition can influence systemic immune tone and epithelial barrier integrity, thereby impacting distal organs such as the lung (8–11). Experimental models have shown that gut dysbiosis can exacerbate pulmonary inflammation by altering cytokine profiles, microbial metabolite levels, and leukocyte trafficking (11–13). Commensal microbes also produce signals that shape hematopoiesis and immune cell development, thereby tuning host susceptibility to allergic and inflammatory disease (14).

Among commensal bacteria, *Bacteroides acidifaciens* (BA) has gained attention for its immunomodulatory properties in mucosal tissues. BA is enriched in various inflammatory conditions and plays context-dependent roles in host immunity (15, 16). In male mice, BA has been shown to enhance gut barrier function by upregulating tight junction proteins and promoting IL-22 production, thereby alleviating disease severity in DSS-induced colitis (17). BA has also been linked to protection against CD95-mediated hepatocyte apoptosis and systemic inflammation (18). In contrast, female mice colonized with BA show exacerbated inflammatory responses, including increased IgA production, T cell polarization, and disrupted barrier function, suggesting a sex-specific effect of this organism on host immunity (19–21). While IgA typically contributes to mucosal defense, excessive or misdirected IgA responses may exacerbate immune-mediated pathology, particularly in the context of Th17-driven inflammation (22, 23).

Sex differences in immunity are increasingly recognized as critical variables in disease susceptibility and treatment response. Hormonal regulation, microbiota composition, and immune cell function differ between males and females, yet most preclinical studies have historically relied on male animals (24–26). Understanding how BA differentially modulates inflammation across sexes may uncover novel mechanisms of microbial-immune interaction and offer new directions for personalized interventions.

In this study, we investigated the role of Bacteroides acidifaciens (BA) in modulating the severity of acute respiratory distress syndrome (ARDS) through the gut-lung axis, with a focus on sex-specific immune responses. Our findings reveal that BA colonization leads to divergent ARDS outcomes in male and female mice, associated with distinct patterns of cytokine expression, epithelial barrier integrity, and mucosal immune activation. Notably, immunoglobulin A (IgA) emerged as a potential mediator of these sex-dependent effects, suggesting a critical role for IgA-driven immune regulation in shaping host responses to microbial signals. These results underscore the importance of host-microbiota interactions in pulmonary inflammation and highlight sex as a key biological variable in immunological research.

## 2 Materials and methods

### 2.1 Animals

Male and female C3H/HeJ mice (8–10 weeks old) were obtained from Jackson Laboratories and housed under specific pathogen-free (SPF) conditions with ad libitum access to food and water. All experiments were conducted in accordance with The Institutional Animal Care and Use Committee (IACUC) guidelines and approved protocols.

### 2.2 Induction of acute respiratory distress syndrome and BA colonization

C3H/HeJ mice (8–10 weeks old) were randomly assigned to four experimental groups: wild-type (WT) control, Bacteroides acidifaciens (BA) only, SEB only, and BA + SEB. Each group consisted of 3–5 mice per sex per experiment. Male data were obtained from two independent biological replicates, and female data from three independent experiments, using separate cohorts. Statistical analyses were performed on the combined datasets from these replicates to ensure adequate power and reproducibility.

BA (DSMZ) was cultured under anaerobic conditions in chopped meat broth (Anaerobe Systems), and bacterial concentration was adjusted to 1 × ^9^ CFU/mL based on optical density, then confirmed via sheep blood agar (SBA) plating. Mice were inoculated via oral gavage with 100 μL of BA suspension in sterile PBS 72 hours prior to SEB exposure. Colonization was confirmed via stool culture and qPCR analysis.

ARDS was induced using staphylococcal enterotoxin B (SEB; Sigma Aldrich). Mice received an intranasal dose of 5 μg SEB in sterile PBS, followed by an intraperitoneal injection of 2 μg SEB two hours later. All mice were monitored and sacrificed 72 hours after SEB challenge for downstream analyses. Clinical parameters and tissue samples were collected for histological, molecular, and immunological evaluations. We acknowledge inherent biological variability in cytokine expression and immune responses in ARDS models and have accounted for this through the use of multiple independent replicates and pooled statistical analyses.

### 2.3 Clinical assessments

Body weight was recorded daily and reported as percent change from baseline. Lung function was assessed using whole-body plethysmography (DSI/Buxco), and included measurements of Penh, pause (PAU), and respiratory frequency (f). Oxygen saturation was measured non-invasively using the MouseOx Plus pulse oximeter (Starr Life Sciences).

### 2.4 Sample collection

At 72 hours post-SEB exposure, mice were euthanized, and samples were collected, including bronchoalveolar lavage fluid (BALF), lung tissue, colon tissue, serum, and stool. BALF was obtained by flushing the trachea with sterile PBS.

### 2.5 Histology and scoring

Lung and colon tissues were fixed in 10% neutral-buffered formalin, paraffin-embedded, sectioned, and stained with hematoxylin and eosin (H&E). Lung injury was scored using the Matute-Bello criteria (27), and colon injury was evaluated using the Cooper scoring system (28).

### 2.6 RNA sequencing and pathway analysis

Total RNA was extracted from lung tissues using the RNeasy Mini Kit (Qiagen) according to the manufacturer’s protocol. RNA purity and integrity were assessed using a NanoDrop spectrophotometer and Agilent 2100 Bioanalyzer, with only samples displaying RNA integrity numbers (RIN) above 8.0 selected for sequencing. RNA library preparation and high-throughput sequencing were performed by Novogene Corporation (Sacramento, CA).

Libraries were constructed using Novogene’s standard protocol for mRNA sequencing, and paired-end 150 bp reads were generated on the Illumina NovaSeq 6000 platform. Raw reads were subjected to quality control using fastp, and clean reads were aligned to the mouse reference genome (GRCm38) using STAR. Gene-level counts were obtained using featureCounts, and differential gene expression analysis was performed with DESeq2. Genes with an adjusted p-value (false discovery rate, FDR) < 0.05 were considered differentially expressed.

To interpret functional implications of the observed gene expression changes, significant differentially expressed genes (DEGs) were uploaded to Ingenuity Pathway Analysis (IPA; Qiagen). Canonical pathway enrichment, upstream regulator prediction, and network analyses were conducted, including comparative assessment of sex-dependent transcriptional responses to BA colonization in the context of SEB-induced ARDS.

### 2.7 Quantitative PCR

Total RNA was extracted from lung and colon tissues using Qiagen RNeasy Micro Kit and converted to cDNA using a reverse transcription kit (Applied Biosystems). Gene expression was assessed via SYBR Green-based qPCR using primers specific for Il17a, Il22, Il1b, Tnfa, and Il6 in the lung. Muc2, Muc3, Cldn2, and Cldn4 expression was determined in the colon. Gene expression was normalized to housekeeping genes and analyzed using the ΔΔCt method.

### 2.8 Enzyme-linked immunosorbent assay

Cytokines IL-17 and IL-22 were measured in BALF using ELISA kits (BioLegend). IgA levels were quantified in both BALF and serum using ELISA kits (Invitrogen). Assays were performed according to the manufacturers’ protocols.

### 2.9 FITC-dextran assay

To assess gut permeability, mice were fasted for 4 hours and orally gavaged with 100 μL of FITC-dextran (4 kDa, 80 mg/mL in PBS). After 2 hours, BALF was collected via tracheal cannulation lavage. BALF fluorescence was measured using a plate reader (excitation 485 nm, emission 528 nm) and compared to a standard curve to calculate concentrations.

### 2.10 Flow cytometry

Lung tissue was digested using collagenase D and DNase I, followed by filtering through a 70 μm strainer to obtain single-cell suspensions. Red blood cells were lysed using red blood cell lysis buffer. Cells were stained using antibody panels as detailed in **Table 1**:

**Table 1:** Flow cytometry panels.

Panel	Marker	Fluorochrome
Lymphoid	CD45	Pacific Blue
	CD4	BV785
	CD8a	Spark Blue 550
	CD19	BV605
	NK1.1	BB700
	CD25	PE-Cy7
	CD44	BUV737
	Foxp3	AF647
	IL-17A	BUV395
Myeloid	CD45	PerCP
	CD11b	AF700
	Ly6G	APC/Fire 810
	Ly6C	BV570
	CD11c	BV650
	SiglecF	Kiravia Blue 520
	CD68	BV421
	CD80	PE-Cy7
	CD206	BV711

Data were acquired using a BD FACSymphony flow cytometer and analyzed using FlowJo software (v10.8).

Flow cytometric analysis was performed using a sequential gating strategy to identify and characterize immune cell populations in lung tissue. Initially, forward scatter (FSC) versus side scatter (SSC) was used to exclude debris and isolate the main cell population. Singlets were then selected based on FSC-A versus FSC-H to eliminate doublets. Leukocytes were identified by positive expression of CD45. Subsequent gating was performed to distinguish lymphoid and myeloid subsets. Lymphoid populations included CD3^+^ T cells, further categorized into CD4^+^,CD25^+^, CD8a^+^, IL-17^+^ (Th17), and Foxp3^+^ (regulatory T cells). Myeloid populations were identified by expression of CD11b and further characterized by Ly6G (neutrophils), CD68 (macrophages), SiglecF (eosinophils or alveolar macrophages), CD206 (M2 macrophages), and CD80 (M1 macrophages).

### 2.11 Statistical analysis

All data were analyzed using GraphPad Prism (v9.0). Differences between groups were assessed using one-way ANOVA with multiple comparisons *post hoc* tests. A p-value < 0.05 was considered statistically significant.

## 3 Results

### 3.1 BA modulates symptoms of SEB-induced ARDS in a sex-dependent manner

**Figure 1:**
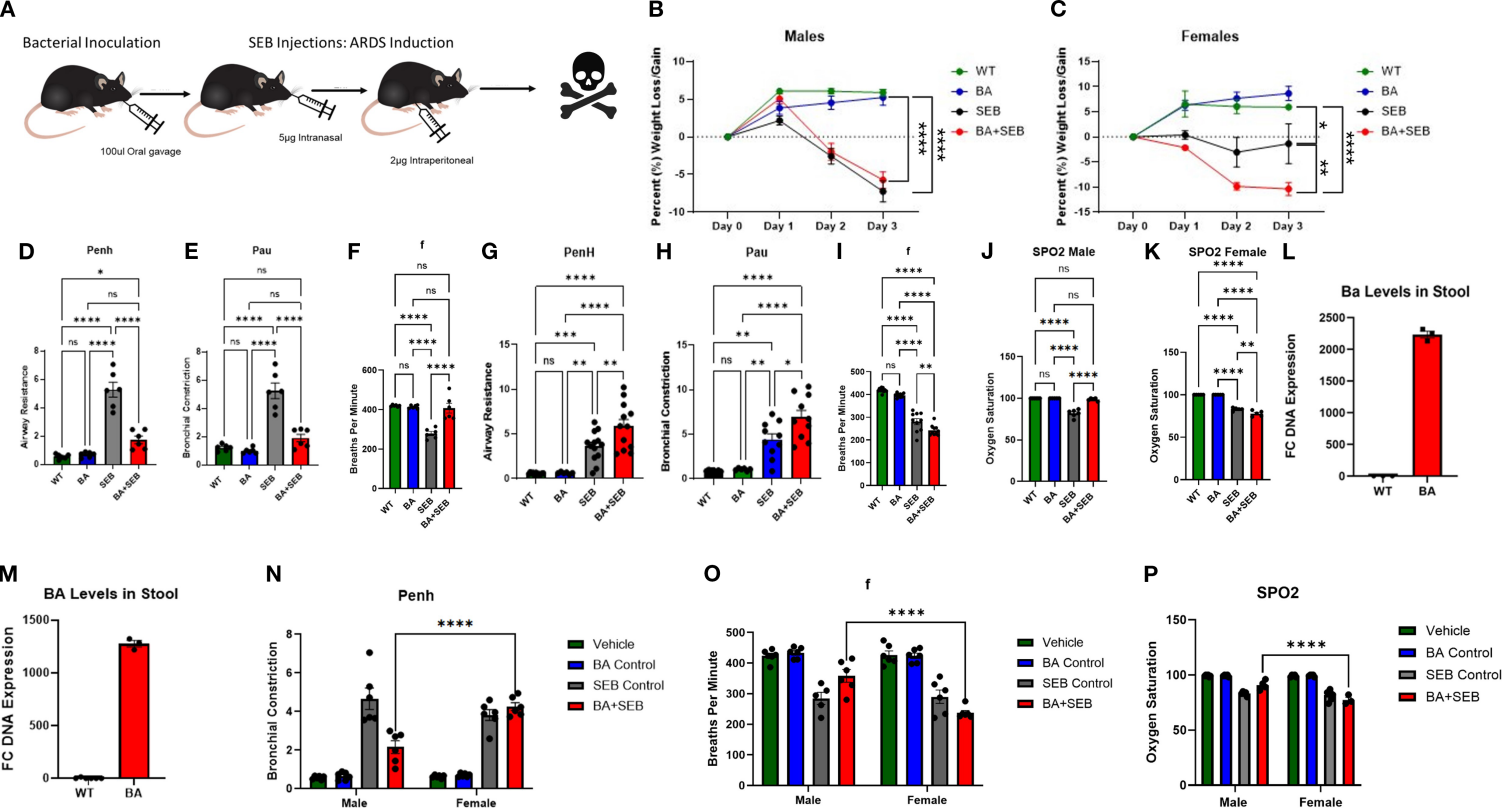
BA modulates symptoms of SEB-induced ARDS in a sex-dependent manner. Male and female mice were colonized with BA or given a vehicle control (PBS) 72 hrs prior to SEB exposure to induce ARDS. **(A)** Schematic overview of experimental design. Percent body weight was measured daily following SEB administration in male **(B)** and female **(C)** mice (males n=6/group, females n=12/group). Male lung function was assessed 72 hours post-SEB using whole-body plethysmography: **(D)** airway constriction (Penh), **(E)** pause (Pau), **(F)** breaths per minute (f). Female lung function was assessed under same parameters: **(G)** airway constriction (Penh), **(H)** pause (pau), **(I)** breaths per minute (f). Male **(J)** and Female **(K)** oxygen saturation was measured via SPO2. DNA was isolated form the stool to validate BA colonization via PCR: **(L)** males, **(M)** females (n=3). Statistical analysis was performed to compare male and female Penh **(N)**, breaths per minute **(O)**, and oxygen saturation **(P)**. Data are presented as mean ± SEM. Statistical analysis was performed using one-way ANOVA or two-way ANOVA with Tukey’s multiple comparison test; *p < 0.05, **p < 0.01, ***p < 1.001, ****p < 0.0001.

To investigate the role of BA in modulating ARDS, male and female mice were orally inoculated with BA or vehicle control prior to SEB administration. A schematic overview of the experimental design is shown in **Figure 1A**. SEB was administered in a dual-dose regimen to induce ARDS, leveraging its ability to act as a superantigen by binding MHC class II molecules and activating a large population of T cells. This activation triggers a cytokine storm that leads to alveolar damage, vascular leak, and impaired gas exchange, modeling the clinical features of ARDS.

Clinical parameters were monitored to assess disease severity. BA+SEB-colonized male mice exhibited slightly reduced weight loss compared to SEB-only controls (**Figure 1B**), while BA+SEB-colonized female mice displayed greater weight loss (**Figure 1C**). Lung function analysis revealed that male mice colonized with BA had significantly lower airway resistance (Penh; **Figure 1D**) and reduced bronchial constriction (Pau; **Figure 1E**), along with increased respiratory rate (f; **Figure 1F**), suggesting improved pulmonary function. In contrast, BA-colonized female mice demonstrated worsened lung function with increased Penh (**Figure 1G**), elevated Pau (**Figure 1H**), and decreased respiratory rate (**Figure 1I**).

Consistent with these trends, BA-colonized males maintained higher oxygen saturation levels (SpO_2_; **Figure 1J**) relative to SEB controls, whereas BA-colonized females experienced greater oxygen desaturation (**Figure 1K**). To confirm successful colonization, PCR analysis of fecal DNA was performed. Both male and female mice inoculated with BA exhibited elevated BA abundance compared to controls (**Figures 1L-M**); however, BA levels were significantly higher in males (**Figure 1L**) than females (**Figure 1M**), despite identical inoculation protocols. This finding suggests sex-dependent differences in gut colonization efficiency, potentially due to immunological or microbiota-related factors.

To further evaluate sex-based differences in lung function and oxygen saturation, two-way ANOVA analyses were conducted comparing male and female mice across treatment groups. While no significant sex differences were observed in the SEB-only or control groups, BA+SEB-treated males and females differed significantly in Penh (**Figure 1N**), respiratory rate (**Figure 1O**), and oxygen saturation (**Figure 1P**). These findings highlight that the sex-dependent divergence in pulmonary outcomes was specifically driven by the presence of BA during ARDS.

Together, these results demonstrate that *BA* colonization significantly alters the clinical course of SEB-induced ARDS in a sex-dependent manner. BA provided a protective effect in male mice, as evidenced by reduced weight loss, improved lung function, and higher oxygen saturation. In contrast, female mice colonized with BA exhibited exacerbated disease, including greater weight loss, worsened lung mechanics, and more pronounced oxygen desaturation.

### 3.2 BA colonization differentially impacts lung pathology in male and female mice

**Figure 2:**
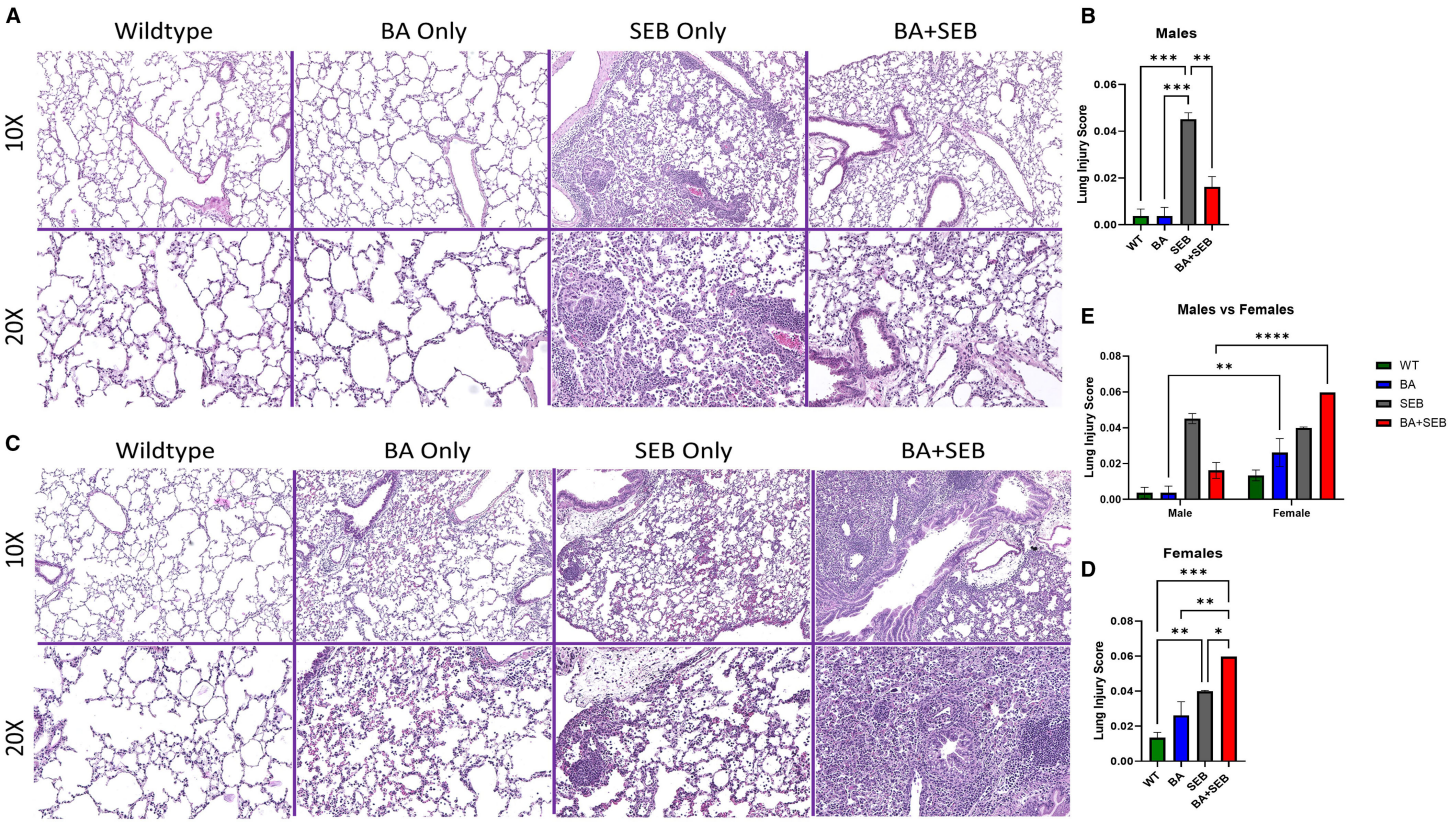
BA colonization differentially impacts lung pathology in male and female mice. Male and female mice were colonized with BA or given a vehicle control (PBS) 72 hrs prior to SEB exposure to induce ARDS. Representative hematoxylin and eosin (H&E)-stained lung sections from male **(A)** and Female **(B)** mice 72 hours post-SEB exposure. Images are shown at 10× (top) and 20× (bottom) magnification. Female BA-colonized mice displayed increased perivascular and alveolar inflammation, alveolar wall thickening, and cellular infiltration compared to controls, whereas male BA-colonized mice exhibited reduced inflammatory features. Quantification of lung pathology scores in female **(C)** and male **(D)** mice based on histological criteria, including neutrophils in alveolar space, neutrophil in interstitial space, alveolar wall thickening, and proteinaceous debris in airspaces (n = 6/group). Direct comparison of male and female lung injury scores was performed **(E)**. Data are presented as mean ± SEM. Statistical significance was determined using one-way ANOVA or two-way ANOVA with Tukey’s multiple comparison; *p < 0.04, **p < 0.02. ***p < 0.0002.

To determine how BA colonization influences lung pathology during SEB-induced ARDS, we performed histological evaluation of lung tissues from male and female mice. In male mice, SEB exposure induced typical ARDS features, including interstitial edema, septal thickening, and leukocyte infiltration. However, BA co-colonization appeared to reduce these effects, with improved preservation of alveolar architecture and diminished inflammatory cell infiltration (**Figure 2A**).To quantify lung injury, we applied the standardized histological scoring system described by Matute-Bello et al. (8), assessing neutrophil accumulation, proteinaceous debris, septal thickening, and hyaline membrane formation. BA-colonized males exhibited significantly lower lung injury scores compared to SEB-only controls, consistent with reduced tissue damage (**Figure 2B**).

In contrast, female mice showed an exacerbated histological response to BA colonization. Compared to SEB-only females, BA+SEB-treated females displayed more extensive alveolar wall thickening, dense neutrophilic infiltration, and increased proteinaceous material within alveolar spaces (**Figure 2C**). High-power fields further revealed greater cellular density, loss of alveolar airspace, and evidence of epithelial remodeling. Quantification of lung injury confirmed this heightened pathology in BA-colonized females, who exhibited significantly higher injury scores than their SEB-only counterparts (**Figure 2D**).

To directly assess sex-dependent differences in lung pathology, we performed a two-way ANOVA comparing lung injury scores across treatment groups between males and females (**Figure 2E**). Significant sex differences were observed within the BA-only and BA+SEB groups, with BA-treated females displaying markedly higher lung injury scores than BA-treated males. These findings support the notion that BA exerts opposing effects on lung tissue integrity depending on host sex.

Together, these findings demonstrate that BA colonization modulates lung histopathology in a sex-dependent manner—mitigating SEB-induced lung injury in males while exacerbating it in females.

### 3.3 BA colonization modulates pulmonary cytokine expression during SEB-induced ARDS in a sex-specific manner

**Figure 3:**
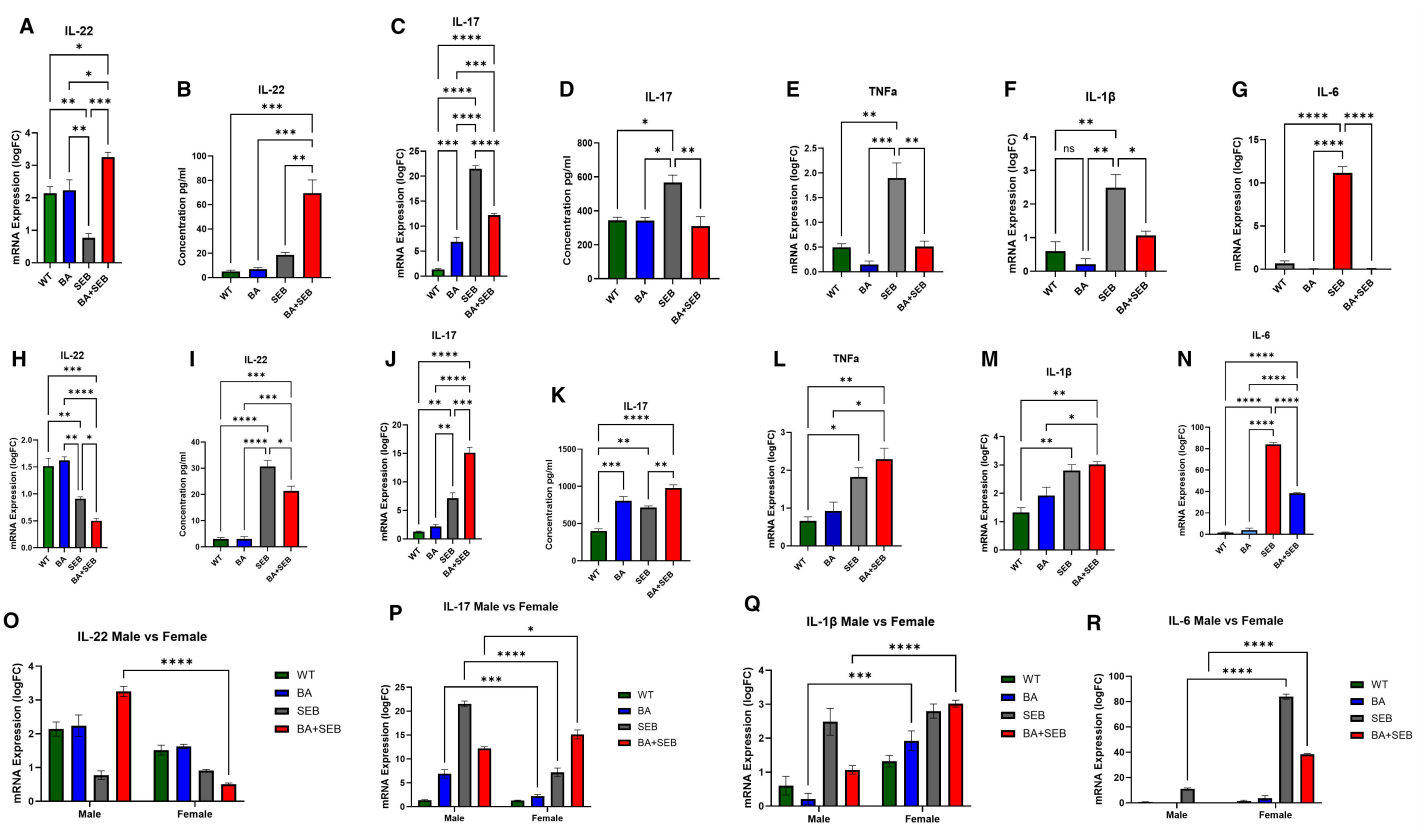
BA modulates lung cytokine responses during SEB-induced ARDS in a sex-dependent manner. Male and female mice (n = 3–5/group) were colonized with BA or given a vehicle control (PBS) 72 hrs prior to SEB exposure to induce ARDS. Cytokines were elevated in the lung tissue or BALF using PCR or ELISA methods. Measurement of IL-22 expression **(A)** and concentration **(B)** in male mice. Measurement of IL-17 expression **(C)** and concentration **(D)** in male mice. Expression of TNFα **(E)**, IL-1β **(F)**, and IL-6 **(G)** in male mice. Measurement of IL-22 expression **(H)** and concentration **(I)** in female mice. Measurement of IL-17 expression **(J)** and concentration **(K)** in female mice. Expression of TNFα **(L)**, IL-1β **(M)**, and IL-6 **(N)** in female mice. Direct comparison of male and female cytokines expression shows significant differences in IL-22 **(O)**, IL-17 **(P)**, IL-1β **(Q)**, and IL-6 **(R)**. Data represent mean ± SEM. Statistical analysis was performed using one-way ANOVA or two-way ANOVA followed by Tukey’s multiple comparisons test. *p < 0.05; **p < 0.01; ***p < 0.001; ****p < 0.0001.

To evaluate the impact of BA on lung immune responses during SEB-induced ARDS, we assessed pro-inflammatory cytokine expression by qPCR and ELISA in male and female mice. In male mice, SEB exposure significantly increased IL-22 and IL-17 expression (**Figures 3A-D**). BA colonization markedly enhanced IL-22 mRNA and protein levels in BA+SEB-treated males compared to all other groups, suggesting a strong BA-driven induction of IL-22. Conversely, IL-17 expression was significantly reduced in BA+SEB males compared to SEB alone, indicating suppression of Th17-associated inflammation. Inflammatory cytokines TNFα, IL-1β, and IL-6 were all elevated in SEB-treated mice but were significantly downregulated in BA+SEB males (**Figures 3E-G**), with IL-6 showing the most pronounced suppression. These results suggest that BA colonization in males dampens key inflammatory pathways while enhancing mucosal regulatory cytokines such as IL-22.

In contrast, female mice displayed an opposing response to BA colonization during SEB-induced ARDS. IL-22 mRNA and protein levels were significantly reduced in BA+SEB females compared to SEB alone (**Figures 3H-I**), suggesting a suppression of this protective cytokine. IL-17 levels remained elevated in BA+SEB females, indicating that BA exacerbates Th17 responses (**Figures 3J-K**). TNFα expression was also significantly increased in the BA+SEB group (**Figure 3L**), while IL-1β trended higher but did not reach statistical significance (**Figure 3M**). Interestingly, IL-6 protein levels were slightly reduced in BA+SEB females compared to SEB alone but remained elevated relative to control groups (**Figure 3N**).

To further investigate sex-specific regulation of inflammatory signaling, two-way ANOVA analyses were performed across treatment groups comparing males and females. IL-22 mRNA expression in BA+SEB-treated males was significantly higher than in BA+SEB-treated females (**Figure 3O**), reinforcing the observation that BA promotes IL-22 signaling preferentially in males. For IL-17, males exhibited higher mRNA levels in the BA and SEB groups, whereas BA+SEB females showed significantly elevated IL-17 compared to their male counterparts (**Figure 3P**), indicating a reversal of inflammatory control in the combined treatment condition. IL-1β mRNA expression was significantly lower in BA- and BA+SEB-treated males relative to females (**Figure 3Q**), consistent with suppressed innate immune activation. Similarly, IL-6 mRNA levels were significantly reduced in SEB and BA+SEB-treated males compared to females (**Figure 3R**), confirming that BA attenuates pro-inflammatory cytokine transcription in a sex-dependent manner.

Overall, these results reveal that BA colonization modulates lung cytokine responses differently in males than females. In males, BA promotes a protective cytokine profile characterized by high IL-22 and suppressed pro-inflammatory signaling, whereas in females, BA reduces IL-22 and enhances inflammatory responses, IL-17 and TNFα production. Two-way ANOVA comparisons further support this sex-specific divergence, highlighting that BA+SEB females mount a more pathogenic cytokine profile across multiple inflammatory pathways.

### 3.4 BA induces sex-dependent changes in pulmonary immune cell populations during ARDS

To investigate how BA modulates pulmonary immune cell composition during SEB-induced ARDS, we performed flow cytometric analysis on lung single-cell suspensions from male and female mice.

**Figure 4:**
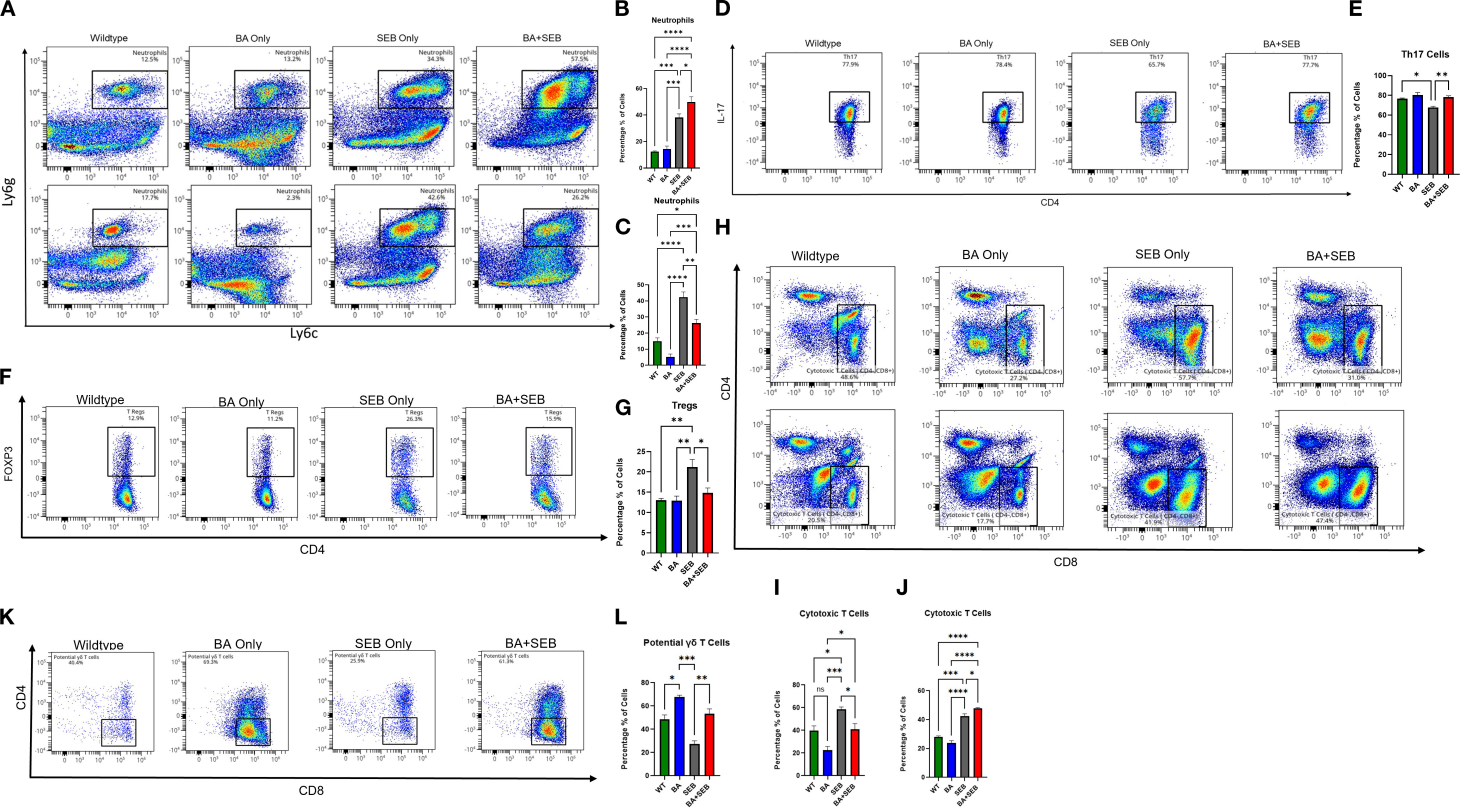
BA alters pulmonary immune cell composition in a sex-dependent manner during SEB-induced ARDS. Male and female mice (n = 3–5/group) were colonized with BA or given a vehicle control (PBS) 72 hrs prior to SEB exposure to induce ARDS. Cell profiling was conducted on lung tissue samples from experimental mice. **(A, B)** Representative flow plots **(B, C)** and percent of lung neutrophils (Ly6G^+^Ly6C^+^) in BA+SEB-treated female and male mice compared to all other groups. Representative flow plots **(D)** and percent of Th17 cells (CD4^+^IL-17^+^) cells in females mice **(E)**. Representative flow plots **(F)** and percent of Treg cells (CD4^+^FoxP3^+^) cells in females mice **(G)**. **(H)** Representative flow plots of female cytotoxic T cells (CD4^-^CD8^+^) for female (top) and male (bottom) samples. **(I)** Percentage of cytotoxic T cells in females. **(J)** Percentage of cytotoxic T cells in males. A population of potential γδ T cells (CD3^+^CD4^-^CD8^-^NK1.1^-^CD19^-^Foxp3^-^CD44^+^CD45^+^) was identified and found to be significantly increased in BA+SEB-treated males, as shown by representative gating **(K)** and quantification **(L)**. Data represent mean ± SEM from n = 3–5 mice per group. Statistical analysis was performed using one-way ANOVA with Tukey’s multiple comparisons test. *p < 0.05; **p < 0.01; ***p < 0.001; ****p < 0.0001.

In female mice, BA colonization significantly altered the recruitment and activation of immune cells. Neutrophils (Ly6G^+^Ly6C^+^) were markedly increased in the BA+SEB group (**Figures 4A** top row-**4B**), indicating enhanced innate immune activation. Similarly, the frequency of Th17 cells (CD4^+^IL-17^+^) was significantly elevated in BA+SEB females (**Figures 4D, E**), consistent with the cytokine data showing sustained IL-17 expression. In contrast, regulatory T cells (CD4^+^Foxp3^+^) were significantly reduced in BA+SEB females relative to SEB-only controls (**Figures 4F, G**), suggesting a shift toward a more pro-inflammatory T cell profile.

Cytotoxic CD8^+^ T cells were also increased in BA+SEB females (**Figures 4H** top row, **Figure 4I**), further indicating an amplified effector T cell response. However, this trend was reversed in male mice, where BA+SEB treatment led to a significant decrease neutrophils (4A bottom row and 4C) and in cytotoxic T cell populations compared to SEB alone (**Figures 4H**, bottom row; **Figure 4J**), highlighting a sex-specific divergence in adaptive immune activation.

Finally, we identified a population of potential γδ T cells (CD45^+^, CD44^+^, CD3^+^, CD4^-^, CD8^-^, NK1.1^-^, CD19^-^, Foxp3^-^) that was significantly increased in BA+SEB male mice (**Figures 4K, L**). These cells may contribute to the altered immune landscape observed in males, potentially mediating protective responses through innate-like mechanisms.

Collectively, these data demonstrate that BA colonization reshapes pulmonary immune cell populations in a sex-dependent manner during SEB-induced ARDS. In females, BA amplifies pro-inflammatory cell subsets—neutrophils, Th17, and cytotoxic T cells—while reducing regulatory T cells, which may drive exacerbated lung injury. In contrast, BA-colonized males exhibit a reduction in cytotoxic T cells and an expansion of potential γδ T cells, supporting a more regulated immune environment associated with improved outcomes.

### 3.5 BA alters gut-lung axis signalling differently in males and females

**Figure 5:**
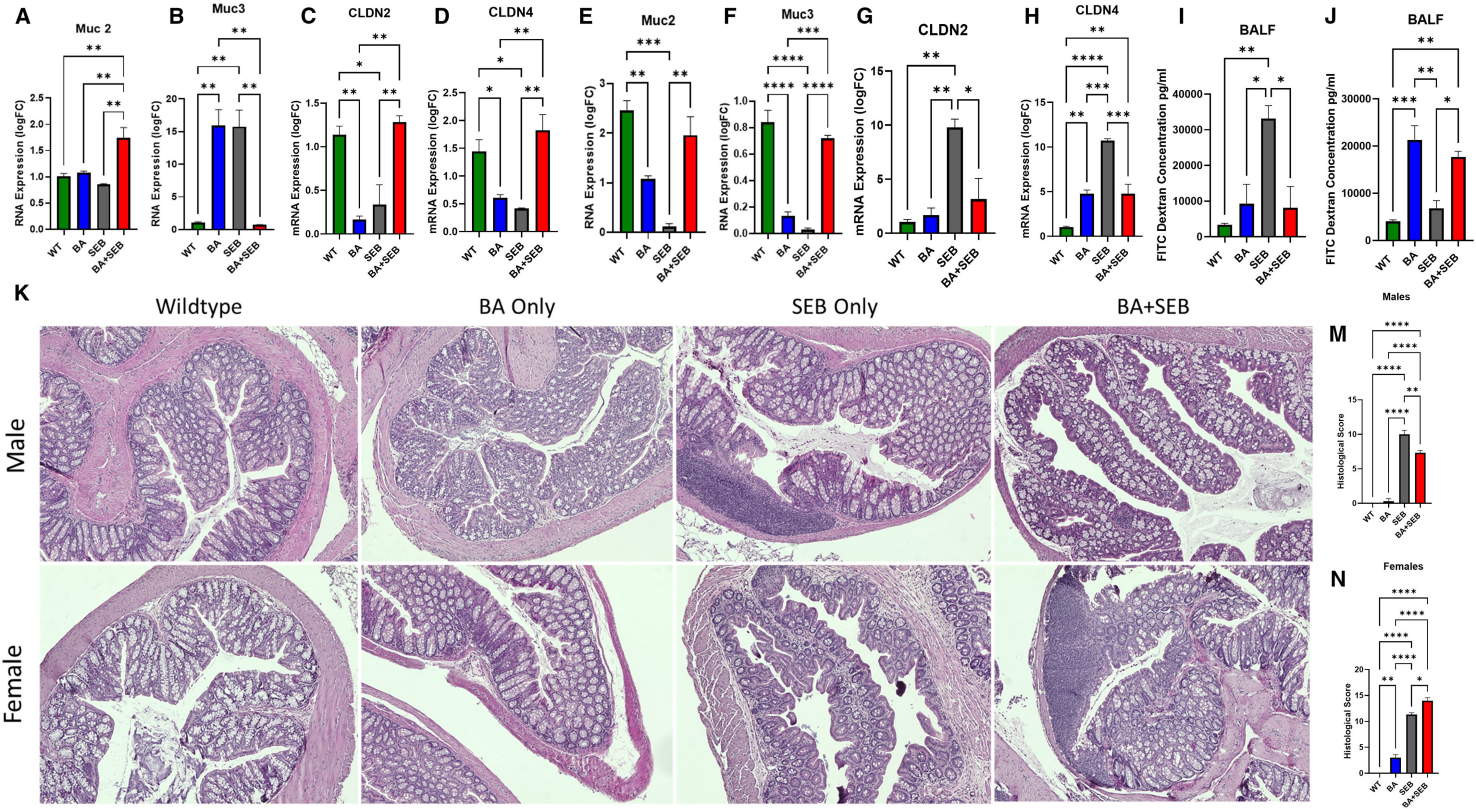
BA alters gut-lung axis signaling differently in males and females. Male and female mice were colonized with BA or given a vehicle control (PBS) 72 hrs prior to SEB exposure to induce ARDS. **(A–D)** Quantitative PCR analysis of colonic gene expression in of Muc2 **(A)**, Muc3 **(B)**, Cldn2 **(C)**, Cldn4 **(D)** in male mice 72 hours post-SEB exposure. **(E-H)** Quantitative PCR analysis of colonic gene expression in of Muc2 **(E)**, Muc3 **(F)**, Cldn2 **(G)**, Cldn4 **(H)** in female mice 72 hours post-SEB exposure. **(I, J)** Intestinal permeability was assessed using FITC-dextran assay for female **(I)** and male **(J)** mice (n = 5/group). **(K)** Representative H&E-stained colonic sections of male (top) and female (bottom) tissues from experimental groups. Histopathological scoring based on epithelial damage, crypt architecture disruption, and inflammatory cell infiltration in male **(M)** and female **(N)** colon tissue samples. Data are shown as mean ± SEM. Statistical comparisons were made using two-way ANOVA with Tukey’s *post hoc* test or unpaired t-tests where appropriate. *p < 0.05, **p < 0.01, ***p < 0.001, and ****p< 0.0001.

To investigate the mechanisms underlying the sex-specific effects of BA on ARDS severity, we assessed transcriptional, functional, and histological changes in the colon. We first examined expression of mucin genes, which are critical for maintaining the intestinal mucus barrier. Muc2 expression was significantly upregulated in male mice colonized with BA when compared to all other groups (**Figure 5A**), indicating a generalized enhancement of mucus layer formation. Muc3, a membrane-associated mucin involved in epithelial signaling, was increased in BA-only and SEB-only groups, but returned to baseline levels in BA+SEB mice (**Figure 5B**). Next, we evaluated the expression of tight junction genes as markers of epithelial barrier integrity. In male mice, BA+SEB treatment led to a significant increase in Claudin-2 (Cldn2) expression (**Figure 5C**), as well as Cldn4 (**Figure 5D**).

In female mice, Muc2 was significantly reduced in SEB-induced ARDs mice compared to controls, but ARDs mice inoculated with BA (BA+SEB) showed significantly higher Muc2 (**Figure 5E**). Muc3 expression in female mice were similar to Muc 2 results (**Figure 5F**). For tight junction proteins, SEB ARDs female mice showed a notable increase in Cldn2 levels, which was reduced with BA colonization (**Figure 5G**), with similar results for Cldn4 (**Figure 5H**).

To evaluate gut-lung axis permeability, we performed a FITC-dextran assay and measured fluorescence from the BALF. Male mice colonized with BA and induced with ARDS showed significantly reduced FITC-dextran levels in the BALF compared to SEB-only controls (**Figure 5I**). Conversely, female BA+SEB mice exhibited increased FITC-dextran levels (**Figure 5J**), indicating compromised barrier function and potential exposure of the lung to gut-derived antigens.

Histological analysis of H&E-stained colon sections further supported these findings. In male mice, BA+SEB colonic tissue exhibited preserved crypt structure, intact epithelium, and minimal immune cell infiltration (**Figure 5K**, top), corresponding to significantly lower histological injury scores (**Figure 5M**). In contrast, BA+SEB-treated female mice displayed disorganized crypts, epithelial damage, reduced goblet cell numbers, and increased cellularity in the lamina propria (**Figure 5K**, bottom), reflected in significantly elevated histological scores (**Figure 5N**).

Collectively, these findings indicate that BA colonization exerts sex-specific effects on intestinal barrier integrity. In males, BA enhances mucin and tight junction expression, reduces gut-lung permeability, and preserves colonic architecture. In females, BA impairs barrier function at multiple levels, contributing to increased gut permeability and mucosal inflammation.

### 3.6 Sex-dependent immune modulation correlates with altered IgA responses

**Figure 6:**
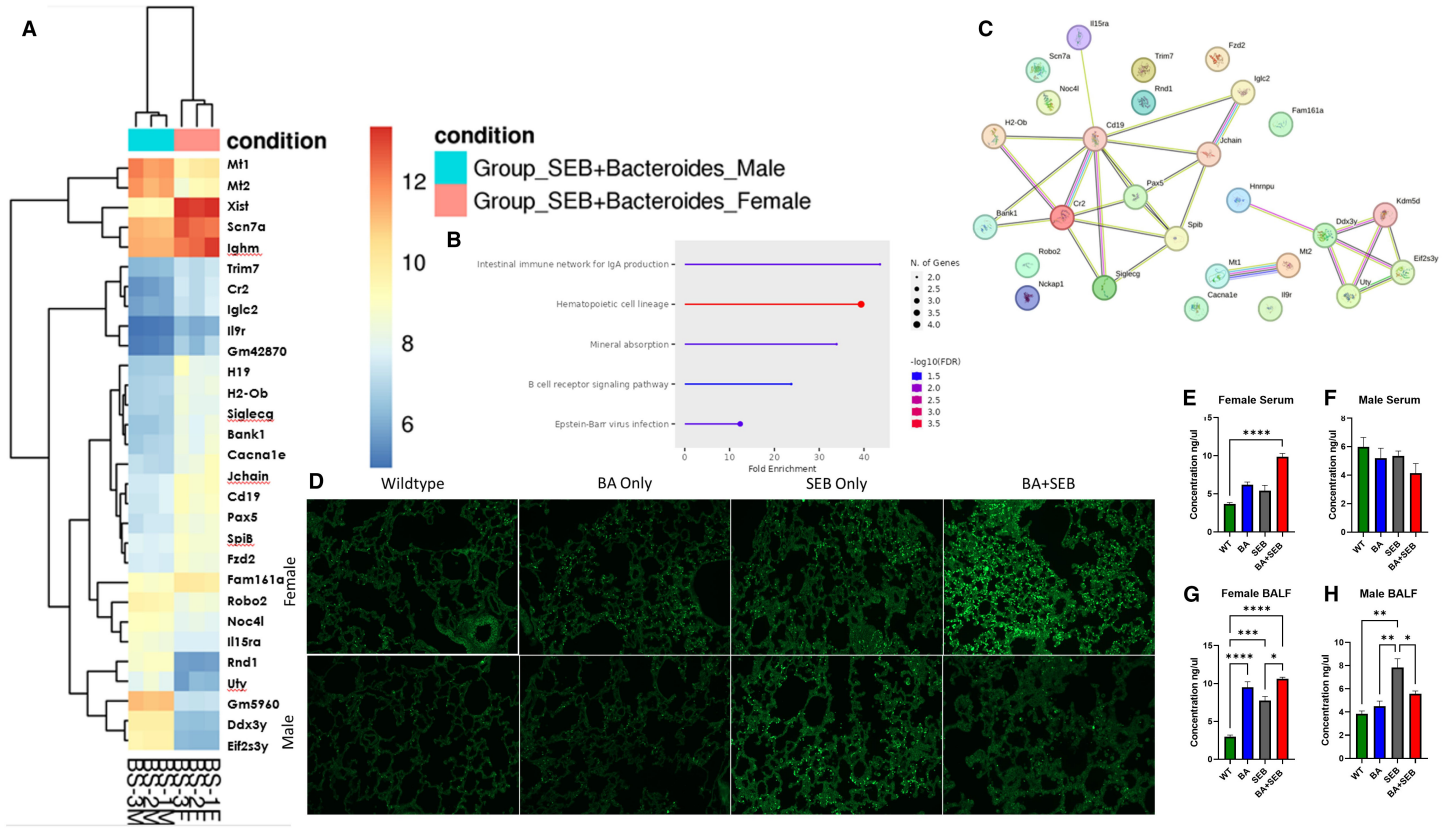
Sex-dependent immune modulation correlates with altered IgA responses. Male and female mice were colonized with BA or given a vehicle control (PBS) 72 hrs prior to SEB exposure to induce ARDS for Bulk RNA sequencing from lung tissues samples. **(A)** Heatmap of Differentially expressed genes (DEGs) from Bulk RNA sequencing of lung tissue of male (blue) and female (red) mice colonized with BA and induced with SEB. **(B, C)** Pathway Enrichment Dot Plot and Protein–Protein Interaction (PPI) Network showing top divergent canonical pathways identified through Ingenuity Pathway Analysis (IPA), highlighting immune signaling, epithelial barrier function, and mucosal immunity pathways enriched in either males or females. **(D)** Representative lung tissue sections stained with fluorescent-labeled IgA to quantitate expression in female (top) and male (bottom) samples. **(E, F)** Concentration of IgA in serum of female **(E)** and male **(F)** samples. **(G, H)** Concentration of IgA in BALF from female **(G)** and male **(H)** samples (n = 5/group). Data are shown as mean ± SEM. Statistical comparisons were made using two-way ANOVA with Tukey’s multiple comparison *post hoc* test. *p < 0.05, **p < 0.01, ***p < 0.001, and ****p< 0.0001.

To investigate transcriptional differences underlying sex-specific immune responses during SEB-induced ARDS, we performed RNA sequencing on lung tissue from BA+SEB-treated male and female mice. A heatmap of differentially expressed genes revealed distinct gene expression patterns between sexes, with a notable cluster of immune-related genes upregulated in females (**Figure 6A**). Ingenuity Pathway Analysis (IPA) was subsequently applied to identify functional pathways associated with these sex-specific transcriptomic changes. Among the pathways that diverged most between males and females were those related to mucosal immunity, B cell signaling, and immunoglobulin production (**Figure 6B**). A focused network map of the intestinal immune network for IgA production highlighted upregulated expression of genes including Cd19, Cr2, Pax5, SpiB, Jchain, Ighm, Bank1, and Siglecg in female samples (**Figure 6C**).

To examine whether these transcriptional differences translated to protein-level changes, IgA immunofluorescence staining was performed on lung tissue sections from BA+SEB-treated mice. Lung sections from female mice showed increased IgA staining compared to males (**Figure 6D**). IgA levels were further quantified in serum and BALF using ELISA. In female serum samples, IgA levels were significantly elevated in the BA+SEB group compared to controls (**Figure 6E**). In male serum, IgA levels showed a slight downward trend in the BA+SEB group, although no significant differences were observed (**Figure 6F**). In the BALF, IgA was increased in BA-colonized females regardless of SEB exposure (**Figure 6G**), whereas in males, IgA levels increased with SEB alone but decreased in the BA+SEB group (**Figure 6H**).

### 3.7 Estrogen suppression attenuates BA-induced exacerbation of SEB-triggered ARDS in females

**Figure 7:**
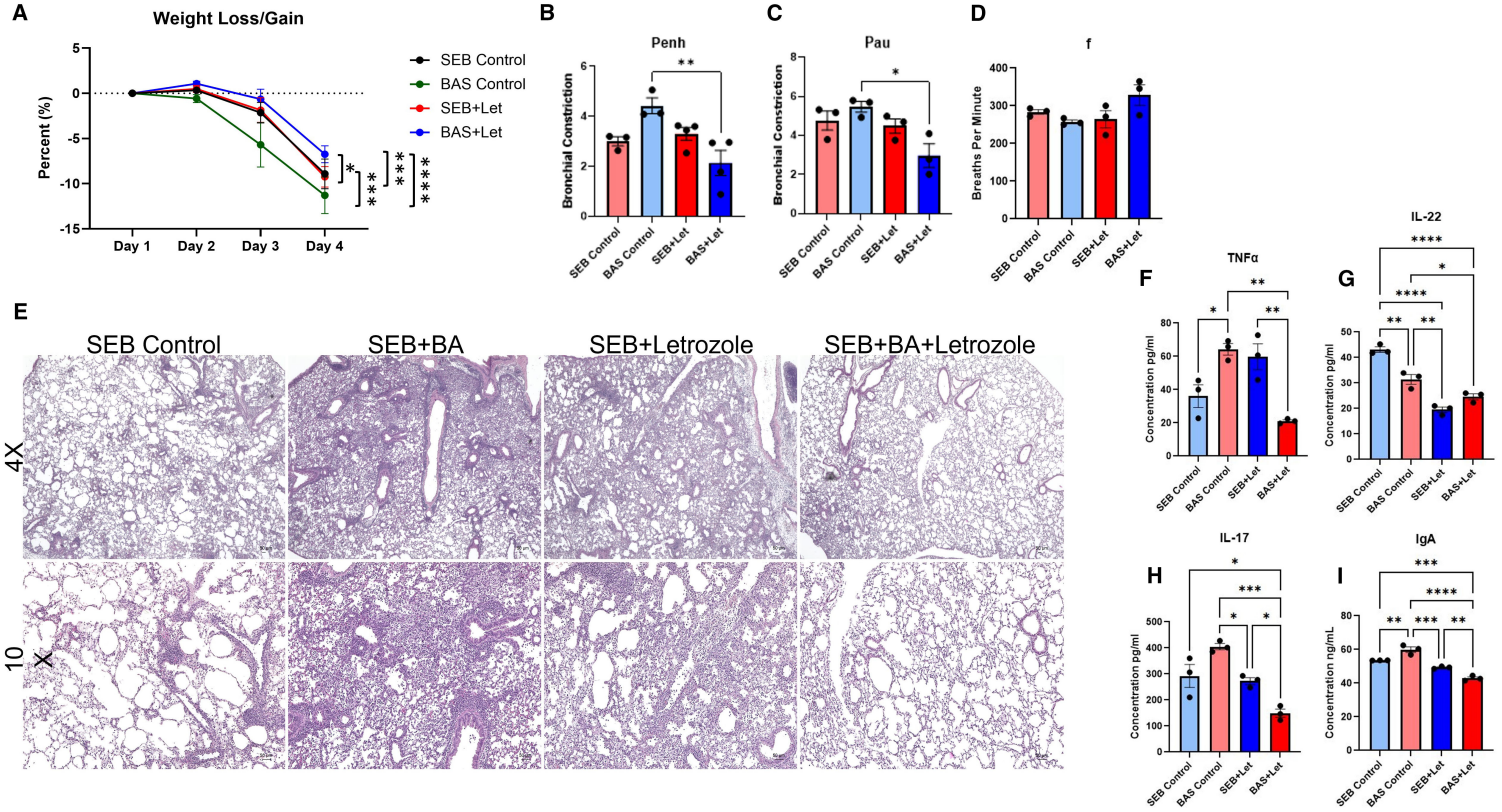
Estrogen inhibition attenuates BA-induced exacerbation of SEB-triggered ARDS in female mice. Female C3H/HeJ mice were treated with Letrozole, a potent aromatase inhibitor, to suppress estrogen production and assess its role in BA-mediated modulation of SEB-induced ARDS. Mice were assigned to four treatment groups: SEB-only, BA+SEB, SEB+Letrozole, and BA+SEB+Letrozole. **(A)** Percent weight change over the course of the experiment. **(B–D)** Lung function parameters assessed 72 hrs post-SEB challenge, including Penh **(B)**, PAU **(C)**, and respiratory frequency **(F, D)**, showing impaired function in BA+SEB mice that was partially rescued by Letrozole treatment. **(E)** Representative H&E-stained lung tissue sections (4X and 10X) showing alveolar architecture disruption and inflammatory infiltrate in BA+SEB mice, with improved tissue integrity in BA+SEB+Letrozole mice. **(F–H)** Cytokine levels in lung tissue quantified by ELISA, including TNFα **(F)**, IL-22 **(G)**, and IL-17A **(H)**. Letrozole treatment decreased TNFα and IL-17A expression, but also reduced IL-22 in both SEB+Letrozole and BA+SEB+Letrozole groups. **(I)** Pulmonary IgA levels measured by ELISA, showing elevated IgA in BA+SEB mice and significant reduction following Letrozole treatment. (n = 4–6/group). Data are shown as mean ± SEM. Statistical comparisons were made using one-way ANOVA with Tukey’s multiple comparison *post hoc* test. *p < 0.05, **p < 0.01, ***p < 0.001, and ****p < 0.0001.

To determine whether estrogen plays a role in the exacerbation of SEB-induced ARDS by BA in female mice, Letrozole—a potent aromatase inhibitor—was administered daily for 7 days prior to SEB challenge and continued throughout the experiment. Mice were assigned to four groups: SEB-only, BA+SEB, SEB+Letrozole, and BA+SEB+Letrozole. Clinical outcomes, lung function, histopathology, and immune responses were assessed 72 hours post-SEB challenge (**Figure 7**).

Vehicle-treated BA+SEB females exhibited significant weight loss compared to SEB-only controls. Letrozole treatment significantly mitigated weight loss in the BA+SEB+Letrozole group, restoring weight maintenance to levels similar to SEB-only controls (**Figure 7A**). Lung function analysis showed elevated airway resistance (Penh) and decreased enhanced pause (PAU) and mid-expiratory flow (f) in BA+SEB mice, consistent with severe pulmonary dysfunction (**Figures 7B–D**). Letrozole treatment significantly improved all three parameters in BA+SEB-treated females, indicating partial recovery of lung function and reduced airway inflammation.

Histological analysis revealed substantial alveolar thickening, inflammatory infiltrate, and structural damage in the lungs of BA+SEB mice (**Figure 7E**). In contrast, BA+SEB+Letrozole mice showed preserved alveolar architecture and reduced immune cell infiltration, supporting a protective effect of estrogen inhibition on lung tissue integrity.

TNFα and IL-17A levels were significantly elevated in BA+SEB mice, reflecting a hyperinflammatory lung environment (**Figures 7F, H**). Letrozole treatment significantly reduced these cytokines in BA+SEB+Letrozole mice, suggesting dampening of inflammatory responses. However, IL-22 levels—which are associated with epithelial repair—were significantly lower in both Letrozole-treated groups (SEB+Letrozole and BA+SEB+Letrozole) compared to untreated controls (**Figure 7G**). These findings suggest that estrogen is necessary for IL-22 production in this context, and that aromatase inhibition blunts this reparative axis, regardless of microbial status.

IgA levels were significantly elevated in the lungs of BA+SEB-treated females, consistent with estrogen-enhanced mucosal immune activation (**Figure 7I**). Letrozole treatment significantly decreased IgA levels in BA+SEB+Letrozole mice, aligning with transcriptomic findings of estrogen-mediated B cell activation and IgA class switching.

Together, these results reveal that estrogen contributes to the BA-driven exacerbation of ARDS in females by amplifying inflammatory cytokine responses and promoting IgA production. Letrozole effectively attenuates weight loss, lung injury, and pro-inflammatory cytokine expression, while reducing IgA levels. However, the concurrent suppression of IL-22 across Letrozole-treated groups highlights a potential trade-off in epithelial protection, suggesting that estrogen may be necessary for optimal IL-22–mediated repair despite its role in driving inflammation. These findings position estrogen as a key regulator of host–microbe interactions in the female lung and underscore the complexity of sex hormone modulation in microbiome-associated inflammatory disease.

## 4 Discussion

**Figure 8:**
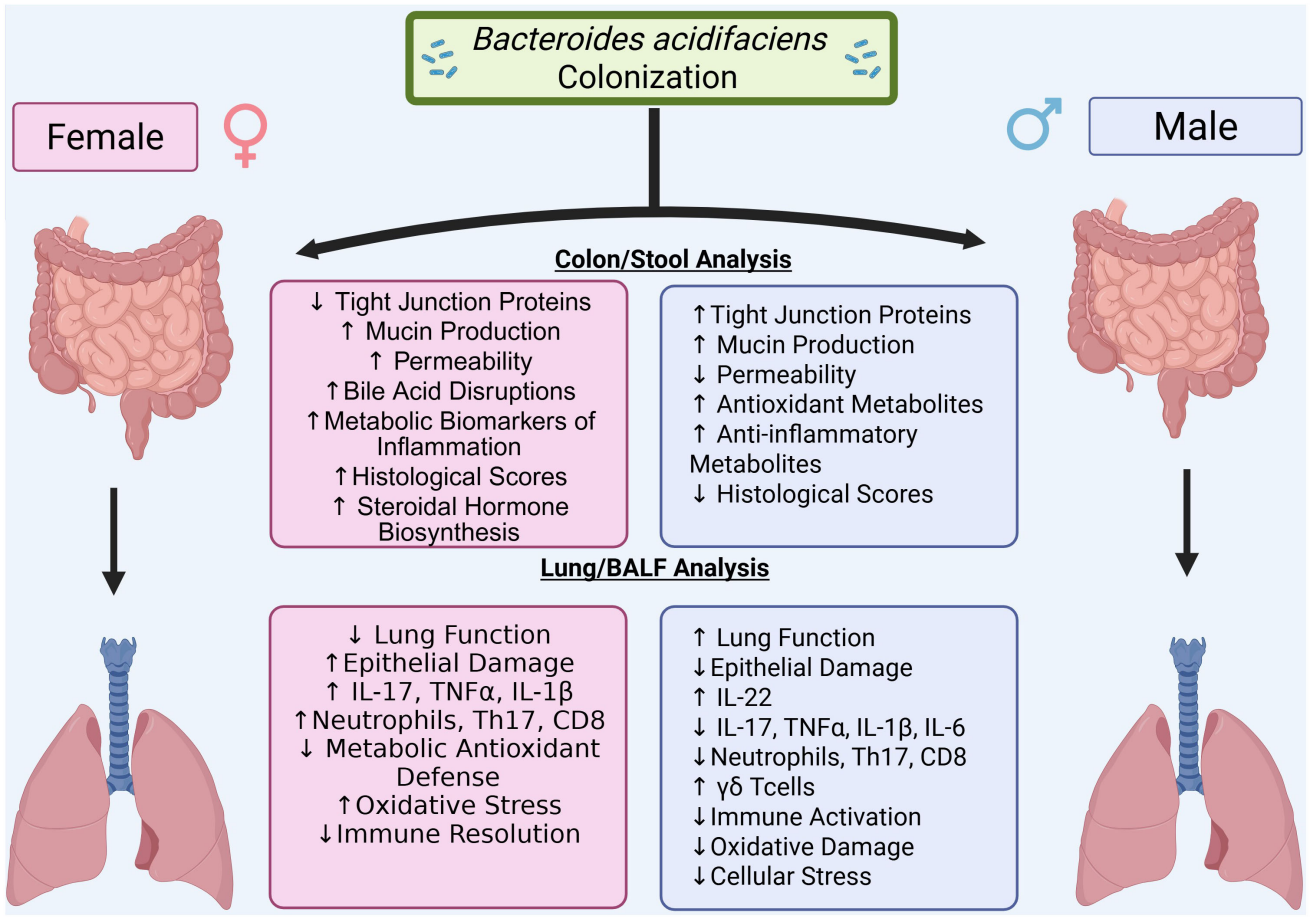
Graphical abstract. Bacteroides acidifaciens (BA) colonization modulates SEB-induced acute respiratory distress syndrome (ARDS) in a sex-dependent manner. In male mice, BA promotes a protective immune response characterized by elevated IL-22 expression, reduced pro-inflammatory cytokines (IL-17A, IL-1β, IL-6, TNFα), improved lung function, and preserved tissue architecture. In contrast, BA exacerbates ARDS in female mice, leading to worsened lung mechanics, greater histological injury, decreased IL-22 expression, and sustained Th17-driven inflammation. These findings highlight the role of host sex in shaping microbiota–immune interactions during pulmonary inflammation.

In the current study, results revealed BA colonization leads to sex-dependent modulation of SEB-induced ARDS, with protective effects in males and disease exacerbation in females. This central finding underscores the critical role of host sex in shaping microbiome–immune interactions. Previous studies have shown that BA can exert beneficial effects in models of colitis, obesity, and liver injury—particularly in male mice (17–19, 29). Our study extends these observations by demonstrating that BA pre-treatment reduces lung inflammation, improves oxygenation, and preserves tissue structure in SEB-induced ARDS, but only in males. Conversely, BA worsened ARDS severity in females, paralleling our prior study, in which BA colonization enhanced inflammation and epithelial damage in female mice with DSS-induced colitis, while not significantly impacting males (21). That study also identified sex-dependent regulation of BA by the aryl hydrocarbon receptor (AhR) in IL-22–producing immune cells, further supporting the idea that host-microbe interactions are fundamentally shaped by sex-specific immune programming. Despite increasing recognition of sex as a biological variable (24–26), few studies explicitly test both sexes in microbiome research as it relates to inflammation. Our findings emphasize that microbial therapies may elicit divergent, even opposing, outcomes depending on host sex, highlighting the need for balanced experimental design in preclinical studies. A conceptual overview of these findings is presented in the graphical abstract (**Figure 8**).

The sex-specific effects of BA on ARDS are reflected in divergent histopathological outcomes in the lung. In male mice, BA reduced alveolar thickening, edema, and immune infiltration which is consistent with a dampened inflammatory response. These findings align with previous reports linking IL-22–driven repair pathways to protection in lung injury models (30, 31). In females, BA exacerbated lung injury, increasing neutrophilic infiltration and proteinaceous debris, consistent with a hyperinflammatory response. Histopathological scoring using the Matute-Bello framework confirmed these differences (28), which mirror the sex-biased outcomes reported in both infectious and sterile models of ARDS (26, 32).

BA modulates lung cytokine profiles in a sex-dependent manner, promoting anti-inflammatory pathways in males and inflammatory skewing in females. In males, BA colonization suppressed classical pro-inflammatory cytokines (TNFα, IL-1β, IL-6, IL-17a) and significantly upregulated IL-22, a cytokine linked to epithelial regeneration and antimicrobial defense (30, 31, 33). These shifts were accompanied by a reduction in CD8^+^ T cells and increased CD45+, CD44+, CD3^+^, CD4^-^, CD8^-^, NK1.1^-^, CD19^-^, Foxp3^-^ cells lacking conventional lineage markers, consistent with γδ T cells. Prior work has shown that γδ T cells play a pivotal role in mucosal repair and IL-22 production during pulmonary and intestinal injury (34–37). In contrast, BA-treated females showed elevated IL-17A and reduced IL-22, with flow cytometry revealing increased CD8^+^ T cells, Th17 cells, and neutrophils, as well as decreased Tregs. This immune profile resembles the pathogenic Th17 expansion observed in chronic mucosal inflammation and autoimmune disorders (38–40).

The gut-lung axis is a key mechanistic bridge in BA’s sex-specific effects, with BA enhancing gut barrier function in males but impairing it in females. In males, BA promoted expression of tight junction proteins (Cldn2 and Cladn4) and preserved colonic architecture, reducing gut permeability and systemic translocation. These findings are consistent with reports demonstrating BA’s ability to fortify mucosal defenses and prevent microbial leakage (17). In females, however, BA failed to upregulate tight junction genes and instead induced epithelial disruption, crypt loss, and mucosal inflammation, which mirrors findings from studies of dysregulated microbiota-induced barrier breakdown under inflammatory stress (23, 41–43). This gut pathology likely amplifies systemic inflammation and lung injury, supporting the growing recognition of gut permeability as a driver of distal organ damage in critical illness (8, 10, 42).

Transcriptomic analysis reveals a sex-divergent immune landscape, with IgA-centered activation in females and regulatory/stress-response pathways in males. Female mice colonized with BA exhibited upregulation of genes involved in B cell differentiation and IgA class switching (*Cd19*, *Cr2*, *Jchain*, *Pax5*, *Ighm*) along with increased pulmonary IgA levels. Although IgA is generally protective in mucosal immunity, its overproduction in inflammatory contexts may lead to immune complex formation, complement activation, and neutrophil recruitment, particularly when paired with Th17 expansion (23, 44, 45). In contrast, BA-colonized males upregulated genes associated with stress tolerance, metabolism, and immune regulation (*Mt1*, *Mt2*, *Il15ra*, *Ddx3y*), suggesting a non-inflammatory program aligned with epithelial maintenance and immune restraint (46–49).

To further investigate the sex-dependent nature of BA’s effects, we used Letrozole, an aromatase inhibitor, to suppress endogenous estrogen production in female mice. Remarkably, estrogen inhibition reversed the deleterious impact of BA in BA+SEB-treated females. Letrozole treatment reduced weight loss, improved lung function, and decreased pro-inflammatory cytokines (e.g., IL-17A, TNFα), while restoring IL-22 levels—mirroring the protective immune profile seen in BA-colonized males. Histological assessment confirmed decreased lung injury in Letrozole-treated females compared to untreated BA+SEB controls.

These findings strongly implicate estrogen as a key mediator of the inflammatory response to BA colonization in females. They also suggest that BA-induced dysregulation of immune responses in the female host is hormonally regulated. Given prior evidence that estrogen can modulate IgA production and transcytosis (50, 51), IL-22 expression (52, 53), and Th17 differentiation (54, 55), our data support a model in which estrogen amplifies BA-driven immune activation and tissue injury. This hormonal control axis may explain why BA elicits such contrasting outcomes in male versus female hosts and highlights estrogen signaling as a potential therapeutic target in microbiome-associated ARDS.

Collectively, our findings establish a mechanistic framework in which BA induces protective immunomodulation in males while promoting IgA-heightened inflammatory pathology in females.

This sex-based dichotomy in BA’s effects underscores a broader principle: commensal microbes can have dual roles in health and disease depending on host context. While BA has been characterized as beneficial in various metabolic and inflammatory models (17–19), most studies to date have been conducted exclusively in male animals, potentially overlooking adverse effects in females. Our data highlight the necessity of sex-specific investigation in microbiome research and caution against generalizing microbial “benefits” without rigorous, context-aware evaluation.

## 5 Limitations and future directions

Several limitations of this study should be acknowledged. First, while our experiments were conducted using separate cohorts across two independent replicates in males and three in females, each group included 3–5 mice, which may limit statistical power for detecting subtle biological effects. We addressed this by pooling data across experiments and focusing on reproducible phenotypic differences; however, future studies with larger cohort sizes will be necessary to enhance statistical robustness and enable more granular subgroup analyses.

Second, our experimental design focused on the functional impact of Bacteroides acidifaciens (BA) in a defined SPF mouse model. While colonization was confirmed via culture and qPCR, we did not perform full community-level microbiome profiling (e.g., 16S rRNA sequencing or metagenomics). This limits our ability to contextualize BA’s effects within the broader gut microbial ecosystem, particularly regarding potential microbial interactions that may modulate disease severity. Ongoing studies in our lab include 16S rRNA sequencing of stool and lung samples to characterize compositional shifts associated with BA colonization and SEB challenge.

Third, the identification of γδ T cells in this study was inferred based on flow cytometry marker exclusion and cytokine production profiles, without the use of TCRγδ-specific antibodies or sequencing. While these data suggest the involvement of γδ T cells—particularly in IL-22–driven protection—definitive confirmation is lacking. Future work should incorporate TCRγδ-specific staining panels, TCR repertoire analysis, or cell sorting followed by IL-22 quantification to validate identity and function.

Additionally, although we observed strong sex-dependent effects, the mechanisms by which BA interacts with host estrogen signaling remain incompletely defined. Letrozole-based estrogen suppression reversed BA-exacerbated ARDS in females, implicating hormonal control; however, the downstream molecular targets of estrogen in this context—such as IgA regulation, Th17 polarization, or barrier dysfunction—require further investigation. Future studies using estrogen receptor or IgA knockout mice along with transcriptomic analysis following hormone modulation could elucidate these pathways.

Finally, while we propose that microbial metabolites or outer membrane vesicles (OMVs) may contribute to BA-mediated immune modulation, we did not directly isolate or characterize these products in the current study. BA-derived OMVs have been shown to influence immune signaling in other disease models (17), and we are currently developing protocols to isolate and profile OMVs from BA-colonized mice under ARDS conditions.

Together, these limitations underscore the need for mechanistic validation, broader microbial context analysis, and hormone-focused studies to fully define the pathways by which BA drives sexually dimorphic outcomes in ARDS. Addressing these gaps will inform the rational design of microbiome-targeted interventions that account for host sex, hormonal status, and microbial context.

## 6 Conclusion

Together, these findings reveal a mechanistic framework in which BA promotes immune homeostasis and epithelial protection in males while triggering IgA- and Th17-mediated inflammation in females—via estrogen-dependent mechanisms. This study underscores a broader principle: commensal microbes can exert highly context-dependent effects based on host sex, hormonal milieu, and immune landscape. While BA is frequently cited as a beneficial microbe, most prior studies have been performed exclusively in male animals. Our findings caution against oversimplified interpretations of microbial “benefit” and underscore the importance of rigorous, sex-balanced experimental design in microbiome research.

## Data Availability

The data presented in the study are deposited in Figshare, DOI: 10.6084/m9.figshare.29815253.v1.

## Supplementary material

Supplementary 1 Flow Cytometry Gating Strategy. Representative gating strategy used to analyze immune cells in lung single-cell suspensions. Initial gates were applied based on forward scatter (FSC-A) versus side scatter (SSC-A) to exclude debris and select cell-sized events. Singlets were isolated by gating FSC-H versus FSC-A to eliminate doublets. CD45^+^ leukocytes were identified by histogram gating on the CD45 marker. CD45^+^ cells were further gated for Ly6g^+^Ly6c^+^ (Neutrophils) and CD3 expression to define total T cells. CD3^+^ T cells were then subdivided into CD4^+^CD8^-^ helper T cells (Tregs and Th17) and CD4^-^CD8^+^ cytotoxic T cells using CD4 and CD8 surface markers. CD3^+^Tells were also subdivided into CD4^-^CD8^-^ and then subdivided again into CD19^-^NK1.1^-^ to identify Foxp3^-^CD44^+^ T cells as potential γδ T cells.

Supplementary 2 Differential gene expression analysis of lung tissue from BA+SEB-treated male and female mice using DESeq2. Transcriptomic profiling was performed on lung tissue from male and female mice colonized with Bacteroides acidifaciens and challenged with SEB (n = 3 per group). **(A)** Principal Component Analysis (PCA) showing separation between male (blue) and female (red) BA+SEB samples along PC1 (80% variance) and PC2 (9% variance). **(B)** MA plot depicting log2 fold changes versus mean normalized counts; red points indicate significantly differentially expressed genes (adjusted p < 0.05). **(C)** Sample-wise boxplot of normalized counts across individual replicates showing consistent distribution of expression. **(D)** Heatmap with hierarchical clustering of the top differentially expressed genes, showing distinct expression patterns between sexes. **(E)** Volcano plot highlighting significantly upregulated genes in males (blue) and females (red), with non-significant genes shown in black. Threshold for significance was set at adjusted p < 0.05 and |log2 fold change| > 1.
