# Supplementary figures and images for: Sex-dependent modulation of acute respiratory distress syndrome by Bacteroides acidifaciens: gut microbiome impact on lung inflammation
Source: Front Immunol. 2025 Sep 22;16:1653309. doi: 10.3389/fimmu.2025.1653309 (PMC12497814; doi:10.3389/fimmu.2025.1653309)

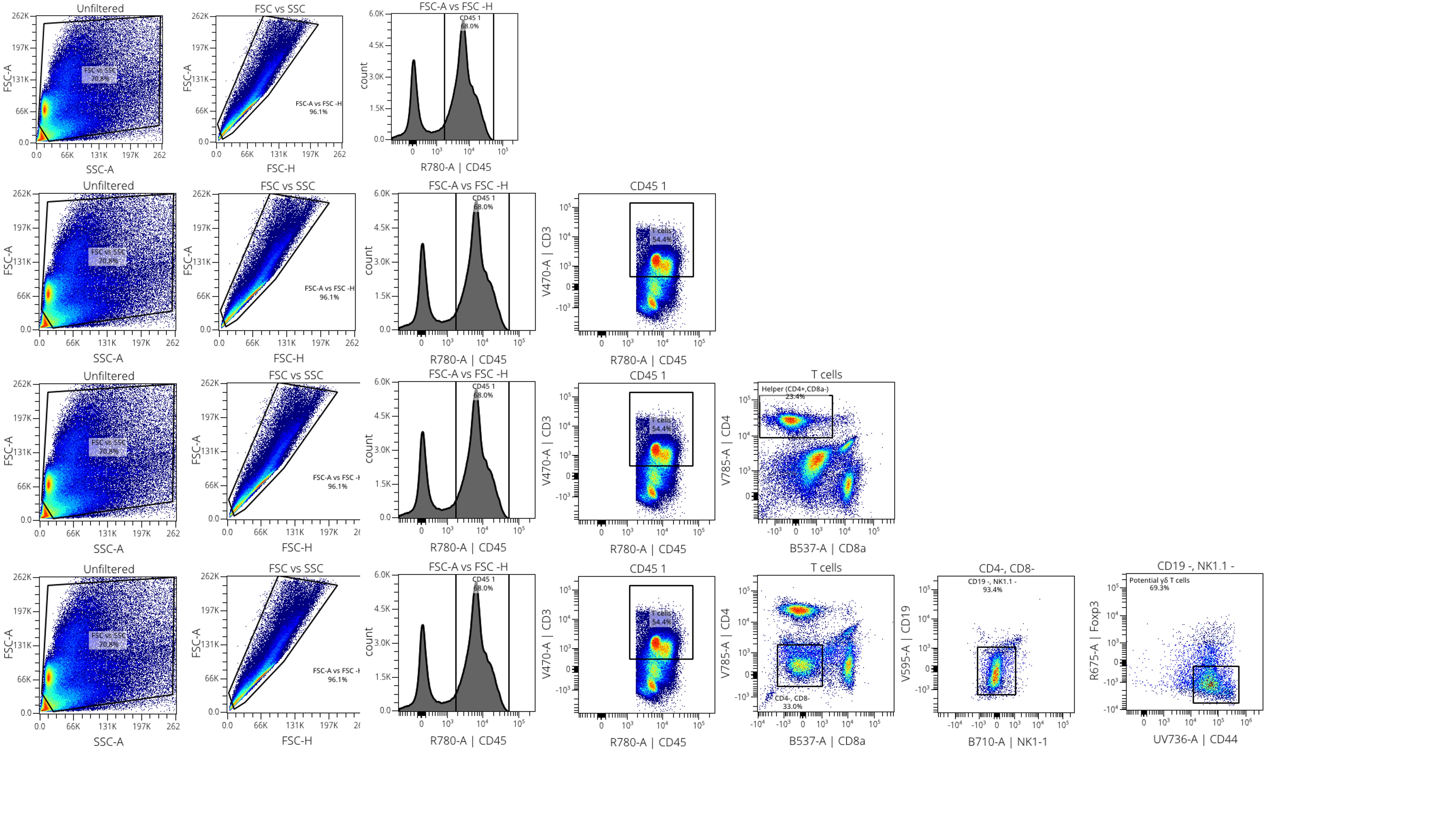

Supplement: Supplementary 1 — Flow Cytometry Gating Strategy. Representative gating strategy used to analyze immune cells in lung single-cell suspensions. Initial gates were applied based on forward scatter (FSC-A) versus side scatter (SSC-A) to exclude debris and select cell-sized events. Singlets were isolated by gating FSC-H versus FSC-A to eliminate doublets. CD45+ leukocytes were identified by histogram gating on the CD45 marker. CD45+ cells were further gated for Ly6g+Ly6c+ (Neutrophils) and CD3 expression to define total T cells. CD3+ T cells were then subdivided into CD4+CD8- helper T cells (Tregs and Th17) and CD4-CD8+ cytotoxic T cells using CD4 and CD8 surface markers. CD3+Tells were also subdivided into CD4-CD8- and then subdivided again into CD19-NK1.1- to identify Foxp3-CD44+ T cells as potential γδ T cells. [file Image1.jpeg]

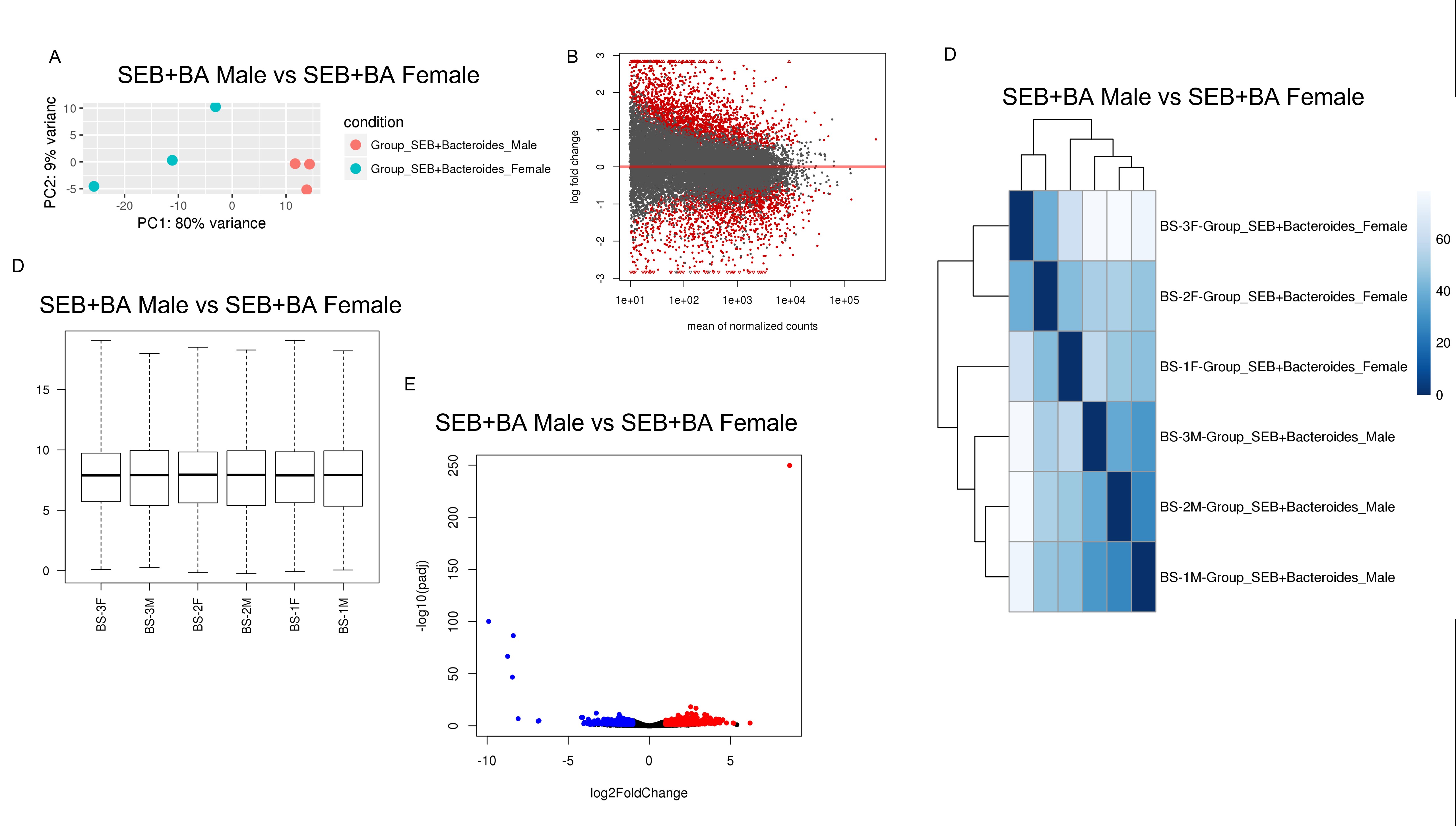

Supplement: Supplementary 2 — Differential gene expression analysis of lung tissue from BA+SEB-treated male and female mice using DESeq2. Transcriptomic profiling was performed on lung tissue from male and female mice colonized with Bacteroides acidifaciens and challenged with SEB (n = 3 per group). (A) Principal Component Analysis (PCA) showing separation between male (blue) and female (red) BA+SEB samples along PC1 (80% variance) and PC2 (9% variance). (B) MA plot depicting log2 fold changes versus mean normalized counts; red points indicate significantly differentially expressed genes (adjusted p < 0.05). (C) Sample-wise boxplot of normalized counts across individual replicates showing consistent distribution of expression. (D) Heatmap with hierarchical clustering of the top differentially expressed genes, showing distinct expression patterns between sexes. (E) Volcano plot highlighting significantly upregulated genes in males (blue) and females (red), with non-significant genes shown in black. Threshold for significance was set at adjusted p < 0.05 and |log2 fold change| > 1. [file Image2.jpeg]
